# [Corrigendum] The p85α regulatory subunit of PI3K mediates cAMP-PKA and retinoic acid biological effects on MCF7 cell growth and migration

**DOI:** 10.3892/ijo.2026.5852

**Published:** 2026-02-02

**Authors:** Caterina F. Donini, Erika Di Zazzo, Candida Zuchegna, Marina Di Domenico, Sonia D'Inzeo, Arianna Nicolussi, Enrico V. Avvedimento, Anna Coppa, Antonio Porcellini

Int J Oncol 40: 1627-1635, 2012; DOI: 10.3892/ijo.2012.1383

Following the publication of the above article, an interested reader drew the authors' attention to the fact, for the wound-healing assay data shown in Fig. 6 on p. 1632, the 'T16 h/p85 WT' and 'T16 h + RA/p85 D' panels appeared to contain an overlapping section, such that data which were intended to show the results of differently performed experiments had apparently been derived from the same original source.

Upon contacting the authors, they realized that Fig. 6 had been inadvertently assembled incorrectly. The revised version of Fig. 6, now showing the correct data for the 'T16 h + RA/p85 D' panel, is shown on the next page. Note that this error did not affect the overall conclusions reported in the study. The authors are grateful to the Editor of *International Journal of Oncology* for allowing them this opportunity to publish a Corrigendum, and all the authors agree with its publication. Furthermore, the authors apologize to the readership for any inconvenience caused.

## Figures and Tables

**Figure 6:**
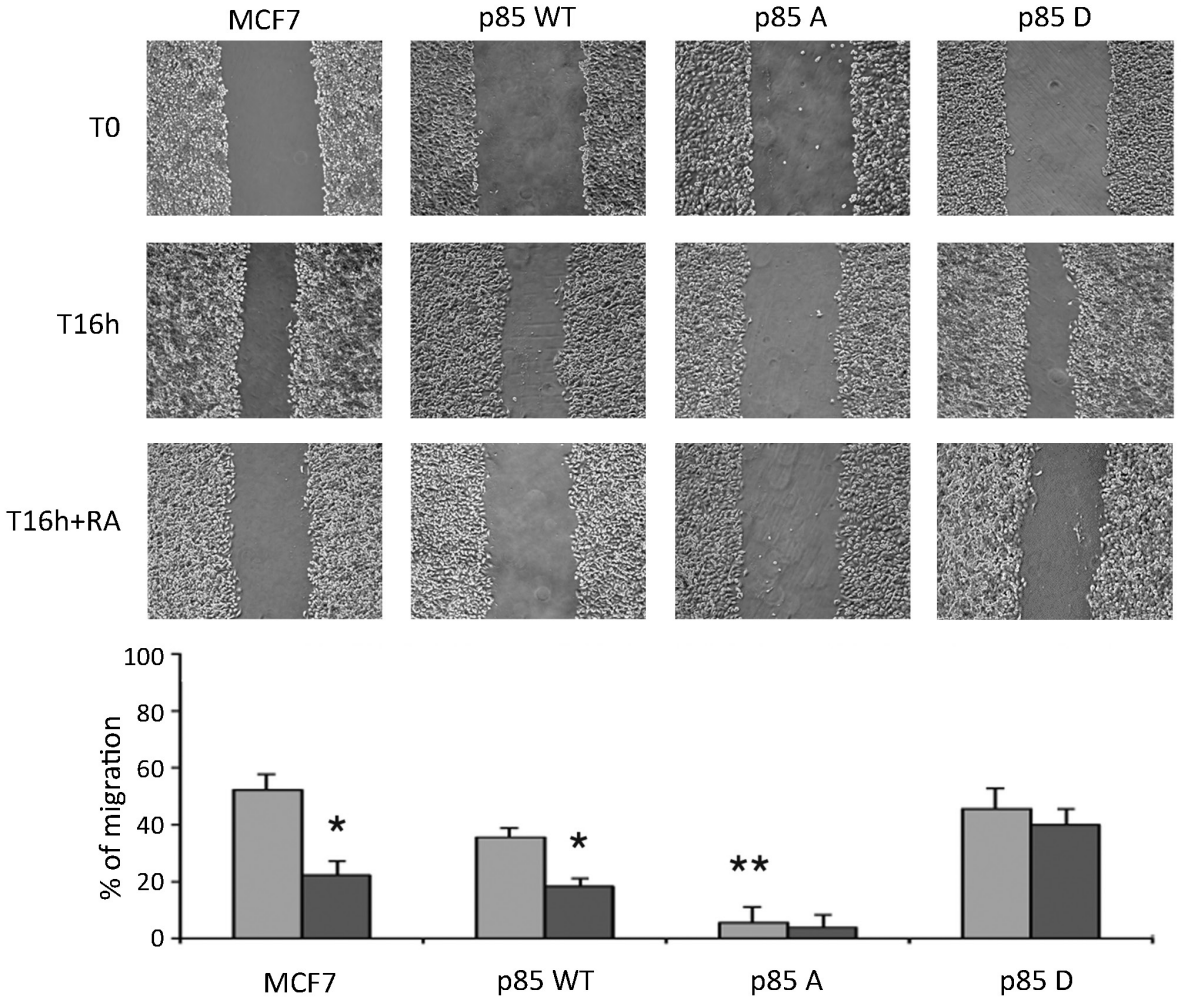
Migratory behaviour of MCF7 transfected cells. Wound-healing assay on MCF7 cells overexpressing p85αPI3K mutants untreated or treated with RA 30 *μ*M for 16 h. Histogram represents quantification of the RA treatment effect on cell motility (% of migration); (^**^P<0.001 comparing p85A vs. empty vector or p85αPI3K wt or p85D; ^*^P<0.05 comparing RA treated vs. basal). Images are representative of three separate experiments performed in triplicate (n=9).

